# Pharmacological interventions targeting β-adrenoceptors in colorectal cancer: an evolving paradigm

**DOI:** 10.1007/s10787-025-01925-5

**Published:** 2025-09-15

**Authors:** Aya Ghosn, Khalil Bassam, Mohammad Al Zein, Suzanne A. Nasser, Gianfranco Pintus, Ali H. Eid

**Affiliations:** 1 Memorial Sloan Kettering, Newyork, NY USA; 2 Cleveland Clinic, Cleveland, OH USA; 3 Keele University, Staffordshire, UK; 4 University of Sassari, Sassari, Italy; 5 Department of Basic Medical Sciences, College of Medicine, QU Health, Qatar University, PO Box 2713, Doha, Qatar

**Keywords:** Malignancy, Chemotherapy, VEGF, COX-2, Cyclin, microRNA, Adrenergic

## Abstract

Colorectal cancer (CRC) is a significant global health challenge, ranking as the third most common cancer and the second leading cause of cancer-related deaths worldwide. Its development is influenced by several risk factors, including smoking, diets rich in red meat, and the effects of stress-related hormones such as epinephrine and norepinephrine. These hormones act through β-adrenergic receptors (β-ARs), which are present on CRC cells and are associated with cancer-promoting processes such as increased cell growth, invasion, blood vessel formation, and accelerated disease progression. Notably, β-ARs blockers have shown potential in slowing CRC progression, pointing to a promising therapeutic strategy. This review explores the main signaling pathways through which β-ARs contribute to cancer development and how various risk factors may influence these mechanisms. We also provide an overview of current preclinical and clinical studies on β-AR blockers in CRC, identify existing gaps in knowledge, and propose directions for future research to optimize therapeutic outcomes.

## Introduction

Colorectal cancer (CRC) is the third most common cancer globally, with 1.93 million new cases reported in 2020, surpassed only by breast and lung cancers(Bray et al. 2018)^,2^. In the same year, CRC was responsible for 916,000 deaths, ranking it as the second leading cause of cancer-related mortality after lung cancer (Bray et al. 2018). CRC often progresses unnoticed, leading to late diagnoses and poorer outcomes.

CRC pathogenesis is heterogenous, often originating from the transformation of various cell types within the colon or rectum. In the majority of instances, the disease begins with the emergence of polyps within these tissues. The subsequent likelihood of malignant transformation is strongly influenced by the specific type of polyp involved (Arnold et al. 2005). For instance, adenomatous polyps, characterized by their dysplastic potential, can progress into adenocarcinomas, which account for approximately 96% of all CRC cases, through a series of chromosomal mutations (Thrumurthy et al. 2016). Conversely, hyperplastic polyps, although typically benign, may occasionally transform into more advanced pre-malignant lesions such as serrated polyps. These serrated polyps are notable for their propensity to accumulate mutations resulting from DNA replication defects and mismatch repair errors, a process known as microsatellite instability, which can ultimately drive the development of CRC (Szylberg et al. 2015).

In most cases, CRC develops in a stepwise manner, starting from the mucosa, the innermost layer of the colon, and advancing outward. As the disease advances, cancer cells may infiltrate blood or lymphatic vessels, facilitating metastasis to distant parts of the body (Aarons et al. 2014). The American Joint Committee on Cancer (AJCC) TNM staging system categorizes its spread, with early stages (0 to IIC) confined to the colon wall and surrounding tissues, while stage III involves lymph node involvement, and stage IV indicates distant metastasis (Weiser 2018). This staging system is essential for assessing disease severity, prognosis, and treatment planning (Weiser 2018). The insidious nature of this progression underscores the critical importance of early detection and intervention. Moreover, understanding the mechanisms driving this stepwise advancement is crucial for developing targeted therapeutic strategies.

### Screening and treatment

Advancements in CRC screening techniques have significantly reduced mortality rates, with a 54% decline reported in 2017 (Siegel et al. 2020). However, age-related disparities persist, as death rates decreased annually by 2.6% for individuals over 55 but rose by 1% per year for those under 55 (Siegel et al. 2020). Approved screening methods include stool-based tests like the fecal immunochemical test (FIT), guaiac-based fecal occult blood test, and stool DNA test, as well as visual procedures such as colonoscopy, CT colonography, and flexible sigmoidoscopy (Wolf et al. 2018).

Treatment strategies for colorectal cancer (CRC) depend on various factors, including individual risk factors, cancer location, cancer stage, and mutational profile, among other factors (Al Zein et al. 2023; Kumar et al. 2023). Surgical resection remains the primary treatment option, while radiation therapy is often used as a neoadjuvant or adjuvant treatment, particularly for rectal cancer, to prevent local recurrence (Kim 2017). Systemic treatments include chemotherapy, targeted therapy, and immunotherapy, with other approaches involving complementary and alternative medicine as well (Athamneh et al. 2020; Benhalilou et al. 2019; Al-Menhali et al. 2015; Al Zein et al. 2023). Common chemotherapeutic agents approved by the FDA include capecitabine, fluorouracil (5-FU), oxaliplatin, and irinotecan (Benson et al. 2013; Younis et al. 2022). Targeted therapy acts on specific cancer genes, proteins, or tumor microenvironment, with limited effects on normal cells. Options include epidermal growth factor receptor (EGFR) inhibitors like cetuximab and anti-angiogenesis drugs such as bevacizumab (Nakayama et al. 2013). Immunotherapy, designed to enhance the immune system's ability to fight cancer, includes pembrolizumab, a PD-1 inhibitor, used to treat unresectable or metastatic CRC with microsatellite instability (MSI-H) or mismatch repair deficiency (dMMR) (Golshani and Zhang 2020).

### Risk factors

There are several risk factors associated with CRC. Approximately 90 percent of CRC cases are diagnosed in people aged 50 and older, making age an important risk factor (Haggar and Boushey 2009; Thrumurthy et al. 2016). A personal history of CRC or adenomatous polyps of the colon increases the risk for the future development of colon cancer (Bonnington and Rutter 2016). Family history of CRC increases the risk by two- to four-fold over that of the general population (Tuohy et al. 2014). It is for this reason that the ACS recommends screening – either colonoscopy or FIT – beginning 10 years or younger than the age of diagnosis of a first degree relative (Wilkinson et al. 2019). Various lifestyle factors also increase the risk of CRC. These include high consumption of red and processed meat (Mejborn et al. 2020), smoking (Amitay et al. 2020) and disruption of the neuroendocrine circadian rhythms, such as when under stress (Zhang et al. 2018). Of note, the adrenergic nervous system plays a major role in the latter effect (Moreno-Smith et al. 2010).

### Adrenergic receptors

Adrenergic receptors (AR) fall into two groups, α and β (Maaliki et al. 2024). The beta adrenergic receptor (β-AR) family comprises three distinct subtypes: β_1_-AR, β_2_-AR and β_3_-AR. These receptors belong to the superfamily of G protein-coupled receptors (GPCR) and are activated by endogenous catecholamines norepinephrine and epinephrine (Velmurugan et al. 2019). Their activation orchestrates a wide array of physiological responses, including cardiovascular regulation, metabolic modulation, and stress adaptation.

**Fig. 1:**
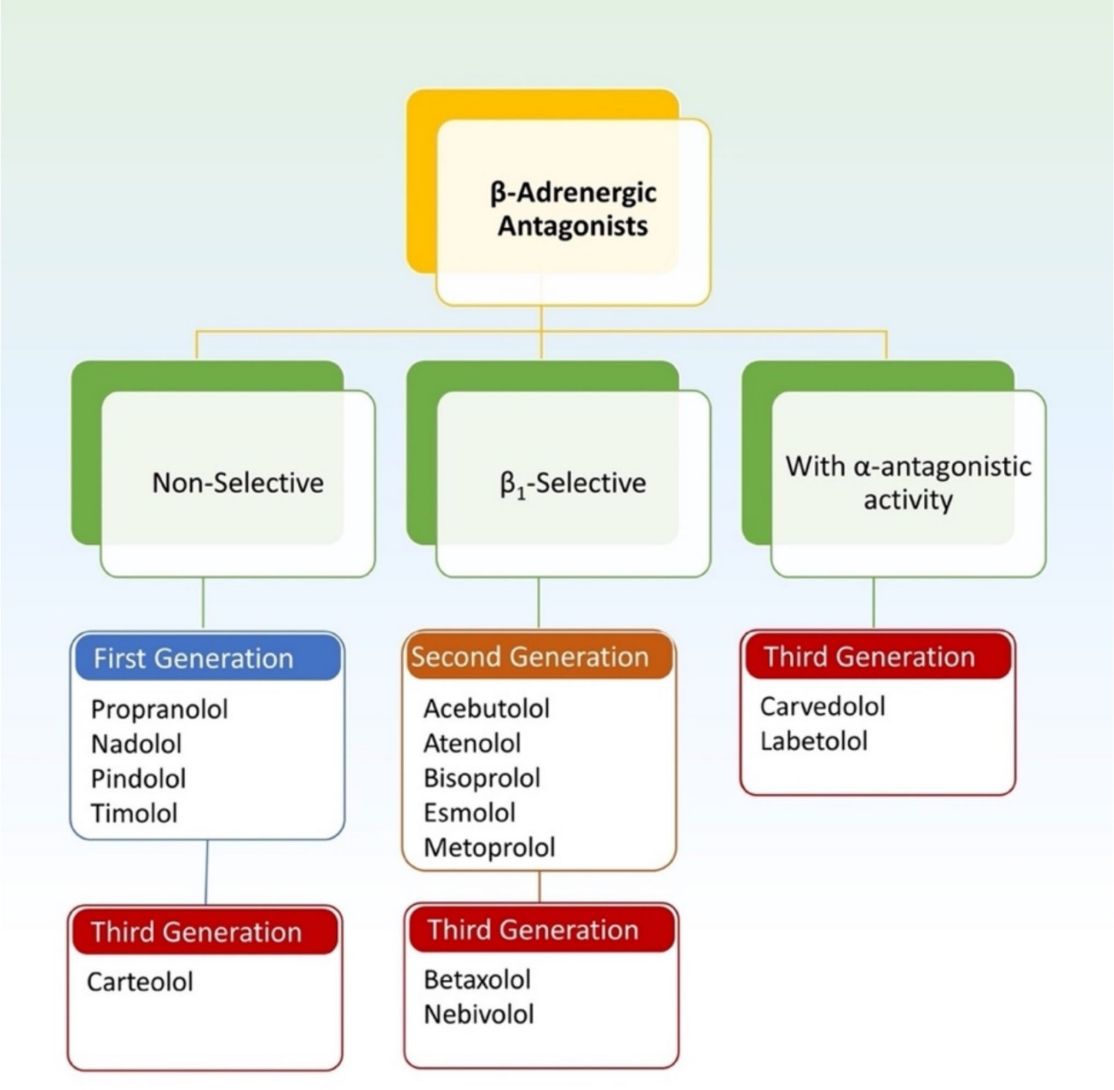
β-adrenergic receptors subtypes. Adrenergic receptors (AR) fall into two groups, α and β. To date, three subtypes of β-ARs have been identified, the β_1_-AR, β_2_-AR and β_3_-AR. β-blockers antagonize the effects of sympathetic nerve stimulation or circulating catecholamines at beta-adrenoceptors. The first generation β-blockers act on both β_1_-AR and β_2_-AR non-selectively. The second generation β-blockers are relatively selective for β_1_-AR at low doses. The third generation β-blockers are non-selective or selective with additional vasodilator actions through blockade of vascular α-AR

β-AR-targeting drugs have evolved over time. First-generation β-blockers act non-selectively on both β1-AR and β2-AR. Second-generation β-blockers, on the other hand, exhibit preferential selectivity for β_1_-AR at lower doses, thereby minimizing adverse effects associated with β_2_-AR blockade. In contrast, third-generation β-blockers represent a distinct pharmacologic class characterized by their unique vasodilatory properties, which confer important hemodynamic advantages and potentially broader therapeutic applications. These agents exert their vasodilatory effects through multiple complementary mechanisms that distinguish them from earlier generations of β-blockers. The primary mechanism involves direct inhibition of vascular alpha adrenoceptors, which counteracts sympathetic vasoconstriction and promotes arterial relaxation. Additionally, this newer generation stimulates the release of nitric oxide (NO) from endothelial cells, activating the cyclic guanosine monophosphate (cGMP) pathway to induce smooth muscle relaxation and further enhance vasodilation. Perhaps most significantly, these agents demonstrate partial agonist activity at vascular β-AR, allowing for balanced receptor modulation that combines β-blockade with intrinsic sympathomimetic activity (Do Vale et al. 2019; Jaillon 1990) (Fig. 1). This additional mechanism renders them particularly advantageous in conditions requiring enhanced vascular relaxation. Furthermore, emerging evidence suggests that β-blockers may confer oncological benefits by improving relapse-free survival and overall survival in patients treated for various malignancies (Phadke and Clamon 2019). Such effects are hypothesized to stem from their ability to counteract the pro-tumorigenic influence of endogenous catecholamines, which act through β-AR signaling pathways implicated in tumorigenesis, angiogenesis, and metastatic progression through intricate signaling pathways (Mravec et al. 2020).

α-adrenergic receptors (α-ARs) are similarly divided into two subtypes: α_1_ and α_2_. α_1_-ARs primarily mediate vasoconstriction of arterial smooth muscle, thereby playing a critical role in cardiovascular homeostasis. Their activation contributes to the maintenance of systemic vascular resistance and blood pressure regulation under sympathetic stimulation. Conversely, α_2_-ARs can exert dual actions: presynaptic α_2-_receptors serve an inhibitory function by suppressing sympathetic activity both centrally and peripherally, whereas postsynaptic α₂-receptors may contribute to vasoconstriction (Lymperopoulos et al. 2013; Duka et al. 2000). Among the α_2_ subtypes, the α_2C_-AR is uniquely implicated in cold-induced vasoconstriction (Haider et al. 2024; Eid et al. 2007), and appears to play a major role in the pathogenesis of Raynaud’s phenomenon (Fardoun et al. 2024; Ture and Lee 2024), primarily through cyclic AMP-mediated mechanisms (Motawea et al. 2013; Motiejunaite et al. 2021).

## β-ARs and CRC

β-ARs have emerged as critical modulators in the pathogenesis of colorectal cancer (CRC) (Chin et al. 2016; Coelho et al. 2015a; Wong et al. 2011). They contribute significantly to tumorigenesis by promoting cellular proliferation, invasion, angiogenesis, and resistance to apoptosis. The mechanisms underlying these effects are multifaceted, involving a network of downstream effectors and signaling pathways. Contextually, β-AR blockade, particularly targeting the β_1_-AR and β_2_-AR subtypes, has been linked with favorable clinical benefits in patients with CRC (Haldar et al. 2020). However, the functional role and therapeutic potential of β_3_-ARs in CRC are not yet fully elucidated. Although there is evidence from at least one study indicating elevated β_3_-adrenergic receptor expression in CRC tissues relative to normal controls, the effects of β_3_ receptor antagonism on tumor growth, progression, and metastatic potential remain inadequately characterized and warrant further investigation (Perrone et al. 2008). This section delineates the primary molecular cascades activated by β-ARs that drive CRC malignancy.

**Fig. 2:**
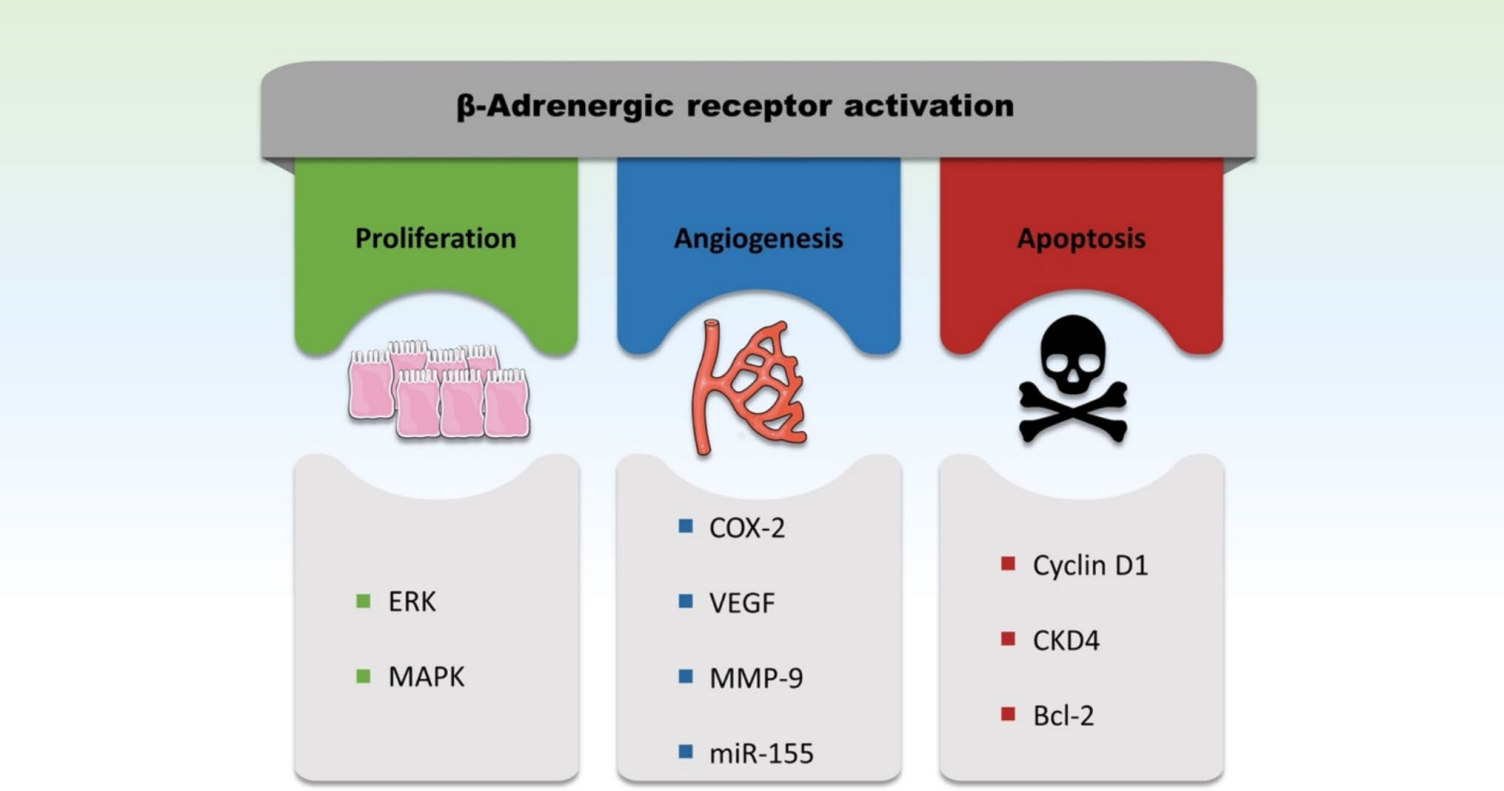
Main downstream pathways of β-AR activation contributing to CRC. β-ARs enhance cell proliferation largely through the activation of ERK1/2, some of the most prominent mitogen-activated protein kinases (MAPK) that drive mitogenic signaling cascades and promote cell cycle progression. β-ARs also promote angiogenesis through the upregulation of cyclooxygenase 2 (COX-2), which in turn increases the expression of vascular endothelial growth factor (VEGF) to stimulate neovascularization and provide tumors with oxygen and nutrients. They are also implicated in invasion through MMP-9, a metalloprotease responsible for the degradation of extracellular matrix proteins. This MMP-9 is well-known to facilitate cancer cell migration, invasion and tissue infiltration as well as metastatic spread. β-ARs also play a role in cellular evasion of apoptosis. Their blockade can induce the upregulation of pro-apoptotic Bcl-2 molecules and downregulation of cyclin D1 and CDK4, ultimately leading to apoptotic death of malignant cells and tumor regression

The mitogenic effects of β-AR activation in CRC are predominantly mediated through the extracellular signal-regulated kinase/mitogen-activated protein kinase (ERK/MAPK) pathway (Schuller 2010; Lin et al. 2013). ERK1 and ERK2, the principal kinases in this cascade, regulate transcription factors that orchestrate cellular proliferation, including neoplastic growth (Keshet and Seger 2010; Flores et al. 2019) (Fig. 2). Epinephrine through activation of β-ARs, robustly induces ERK1/2 phosphorylation, thereby activating this pathway and driving tumor cell proliferation.(Lin et al. 2013). Importantly, β-AR antagonists such as atenolol (β1-selective) and ICI 118,551 (β_2_-selective) inhibit ERK1/2 phosphorylation both in vitro and in vivo, effectively abrogating epinephrine-induced mitogenesis(Lin et al. 2013). These findings underscore the pivotal role of β-ARs in CRC cell proliferation and highlight their potential as therapeutic targets.

Beyond proliferation, β-ARs facilitate CRC invasion and angiogenesis through the upregulation of key mediators such as cyclooxygenase-2 (COX-2), vascular endothelial growth factor (VEGF), matrix metalloproteinase-9 (MMP-9), and nuclear factor kappa B (NF-κB). COX-2 is an inducible isoform of the cyclooxygenase enzyme that is upregulated during inflammation and certain malignant processes. Its role in CRC pathogenesis has been extensively documented, with its expression correlating with poorer prognoses(Wang and Dubois 2010; Sheng et al. 2020; Hidalgo-Estevez et al. 2020; Ogino et al. 2008). COX-2 exerts its effects by cleaving arachidonic acid (AA) into various prostanoids, including prostacyclin, thromboxane A₂, and prostaglandin E_2_ (PGE_2_), the latter of which mediates the pro-inflammatory and carcinogenic effect of COX-2 (Wang and Dubois 2010; Wang et al. 2004). Interestingly, upregulation of COX-2 in CRC has also been linked to an increased expression of VEGF which promotes vascularization and angiogenesis (Sheng et al. 2020; Seno et al. 2002). MMP-9, on the other hand, is a metalloprotease responsible for the degradation of extracellular matrix proteins and promoting cancer invasion and neovascularization (Huang 2018) (Fig. 2).

The interplay between catecholamines and key pro-malignant drivers in CRC is well-characterized, revealing a multifaceted role for β-AR signaling in tumor progression. For instance, epinephrine robustly induces the expression and activation of COX-2 and VEGF (Wong et al. 2011; Muthuswamy et al. 2017) These effects are mediated through β-AR-dependent pathways, as demonstrated by the ability of β_1_- and β_2_-selective antagonists (atenolol and ICI 118,551, respectively) to suppress COX-2 and VEGF upregulation in HT-29 CRC cells ^36^. Similarly, epinephrine enhances matrix metalloproteinase-9 (MMP-9) secretion (Mehedinteanu et al. 2021), facilitating extracellular matrix degradation and tumor invasion—a process reversible upon β2-AR blockade.

Epinephrine further exacerbates CRC development and progression by modulating the CEBPB/TRIM2/p53 signaling pathway (Zhou et al. 2022). In mouse models of chronic stress, animals exposed to stress develop a greater number of AOM/DSS-induced colonic tumors compared to controls. This effect is associated with elevated serum epinephrine levels, which promote tumorigenesis and metastasis through epithelial–mesenchymal transition (EMT) and the generation of cancer stem cells (CSCs). Mechanistically, epinephrine induces the expression of CEBPB, a transcription factor that upregulates TRIM2, which in turn facilitates the ubiquitination and destabilization of p53, thereby impairing its tumor-suppressor activity (Zhou et al. 2022). Many of these effects have been reversed by β_2_-AR antagonists (Wong et al. 2011), suggesting that the epinephrine-induced invasion and angiogenesis is mediated by β_2_-ARs. epinephrine enhances stemness, fostering a subpopulation of therapy-resistant cancer stem cells(Zhou et al. 2022), and that ICI 118,551 suppresses stress-induced tumor growth (Guan et al. 2023). Notably, other studies have confirmed that epinephrine enhances cancer stem cell features through activating transcription factor 1 which integrates adrenergic signaling and stemness, while also fostering resistance to conventional therapies. Thus, targeting β_2_-AR represents a promising strategy for mitigating stemness-driven malignancy and enhancing therapeutic outcomes in CRC patients.

**Fig. 3:**
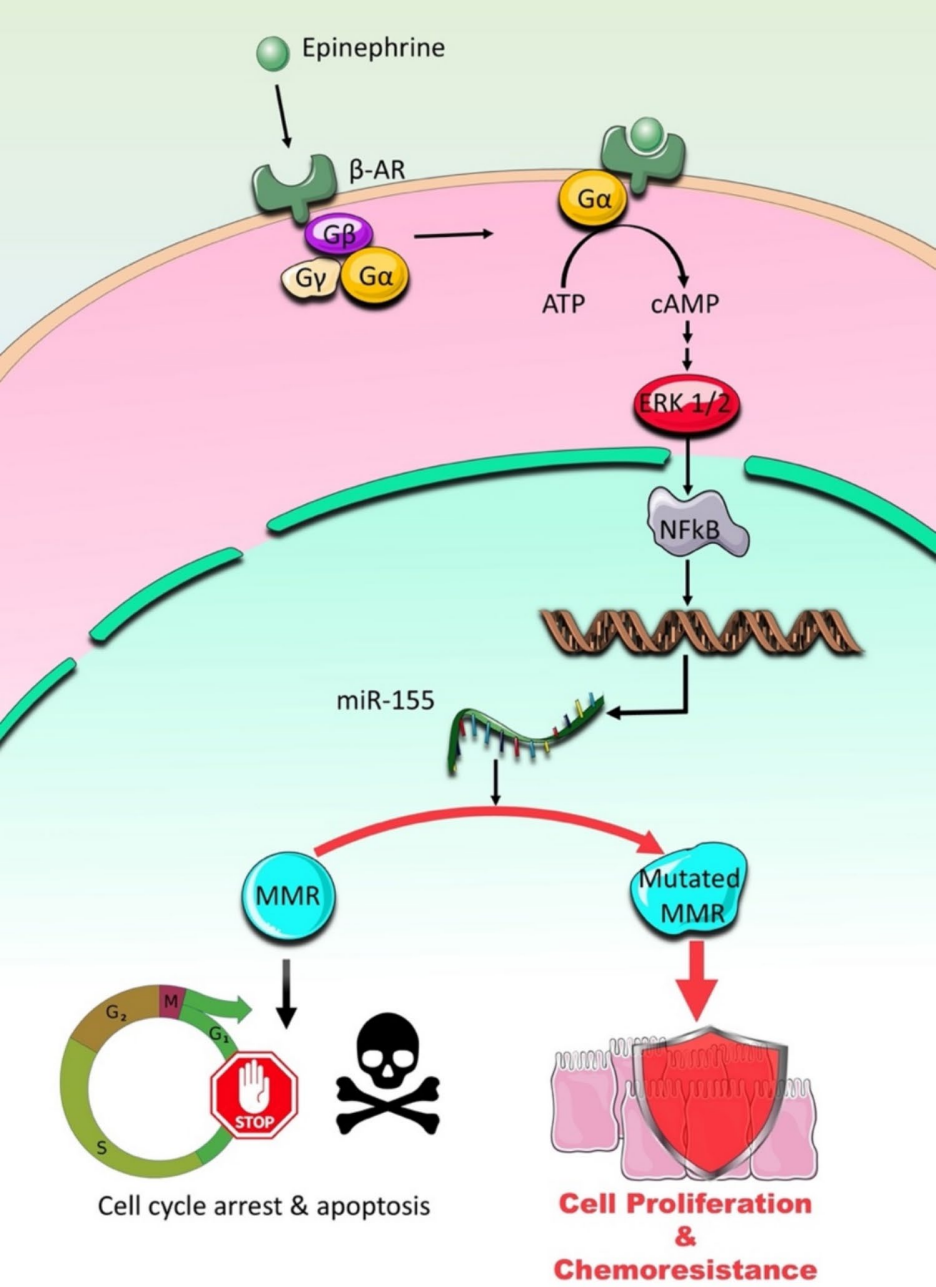
β_2_-ARs implication in CRC proliferation and chemoresistance. β-ARs, including β_2_-ARs, are GPCRs. Upon activation such as by catecholamines, β_2_-AR signaling induces the phosphorylation and subsequent activation of the extracellular signal-regulated kinases (ERK1/2) through canonical MAPK pathway signaling. Activated ERK1/2 then regulate the transcription factor NFkB, leading to increased expression of miR-155, a microRNA that functions as an oncomiR in CRC. miR-155 overexpression is mechanistically linked to mutations and dysfunction in mismatch repair (MMR) proteins, which are essential for maintaining genomic stability through DNA repair processes and cell cycle checkpoint regulation. MMR deficiency allows CRC cells to accumulate genetic mutations and proliferate despite significant DNA damage, thereby promoting both chemoresistance to conventional therapies and sustained tumor cell proliferation through evasion of DNA damage-induced cell cycle arrest

The NF-κB transcription factor plays a pivotal role in regulating genes involved in innate and adaptive immunity, as well as tumor progression (Dorrington and Fraser 2019) (Fig. 3). One of its key downstream targets, microRNA-155 (miR-155), is an oncogenic molecule that has been extensively implicated in colorectal cancer (CRC) pathogenesis (Gao et al. 2018; Hu et al. 2014; Liu et al. 2014; Ling et al. 2013; Higgs and Slack 2013). miR-155 exerts profound effects on proliferation, invasion, chemoresistance, and immune evasion in CRC, making it a critical mediator of malignancy.

miR-155 overexpression is closely associated with chemoresistance in CRC. This microRNA is upregulated by NF-κB activation, which is itself induced by epinephrine during stress responses. Elevated miR-155 levels interfere with mismatch repair (MMR) proteins, which are essential for correcting single nucleotide mismatches and small insertion-deletion errors in DNA (Valeri et al. 2010). These proteins play a critical role in repairing single nucleotide and small insertion-deletion mismatches, recognizing DNA lesions, and triggering cell cycle arrest or apoptosis (Li et al. 2016). Dysfunctional MMR proteins allow cancer cells to proliferate despite accumulating DNA damage caused by chemotherapeutic agents, rendering these cells resistant to treatment. (Cercek et al. 2020). Interestingly, pre-treatment of 5-FU-resistant CRC cells with propranolol (a β-AR blocker) in combination with metformin has been shown to restore sensitivity to 5-FU both in vitro and in vivo (Anselmino et al. 2021). This suggests that β-AR blockade may disrupt the NF-κB/miR-155 axis and enhance chemotherapy efficacy, and that β-blockers may potentially improve overall survival, given that chemoresistance is associated with low survival rates of CRC patients (Anselmino et al. 2021). However, further studies are needed to elucidate whether β-AR antagonists directly downregulate miR-155 expression or act through alternative mechanisms to sensitize chemoresistant CRC cells.

Beyond its role in chemoresistance, NF-κB also plays a role in the immune escape mechanisms of CRC. Clinically, NF-κB subunit 2 (NF-κB2) is upregulated in patients with CRC liver metastasis and plays a central role in suppressing anti-tumor immunity. On a molecular level, NF-κB2 promotes CD8 + T-cell exhaustion—a state of dysfunctional anti-tumor immunity—and upregulates PD-L1 expression on tumor cells. This immunosuppressive milieu facilitates tumor progression and blunts responses to immunotherapy. Intriguingly, pharmacological inhibition of NF-κB2 (e.g., via Rg5) enhances the efficacy of PD-1 checkpoint blockade, suggesting that β-AR antagonists—by indirectly suppressing NF-κB2—could synergize with immunotherapies to overcome immune evasion (Zhang et al. 2024a).

**Fig. 4:**
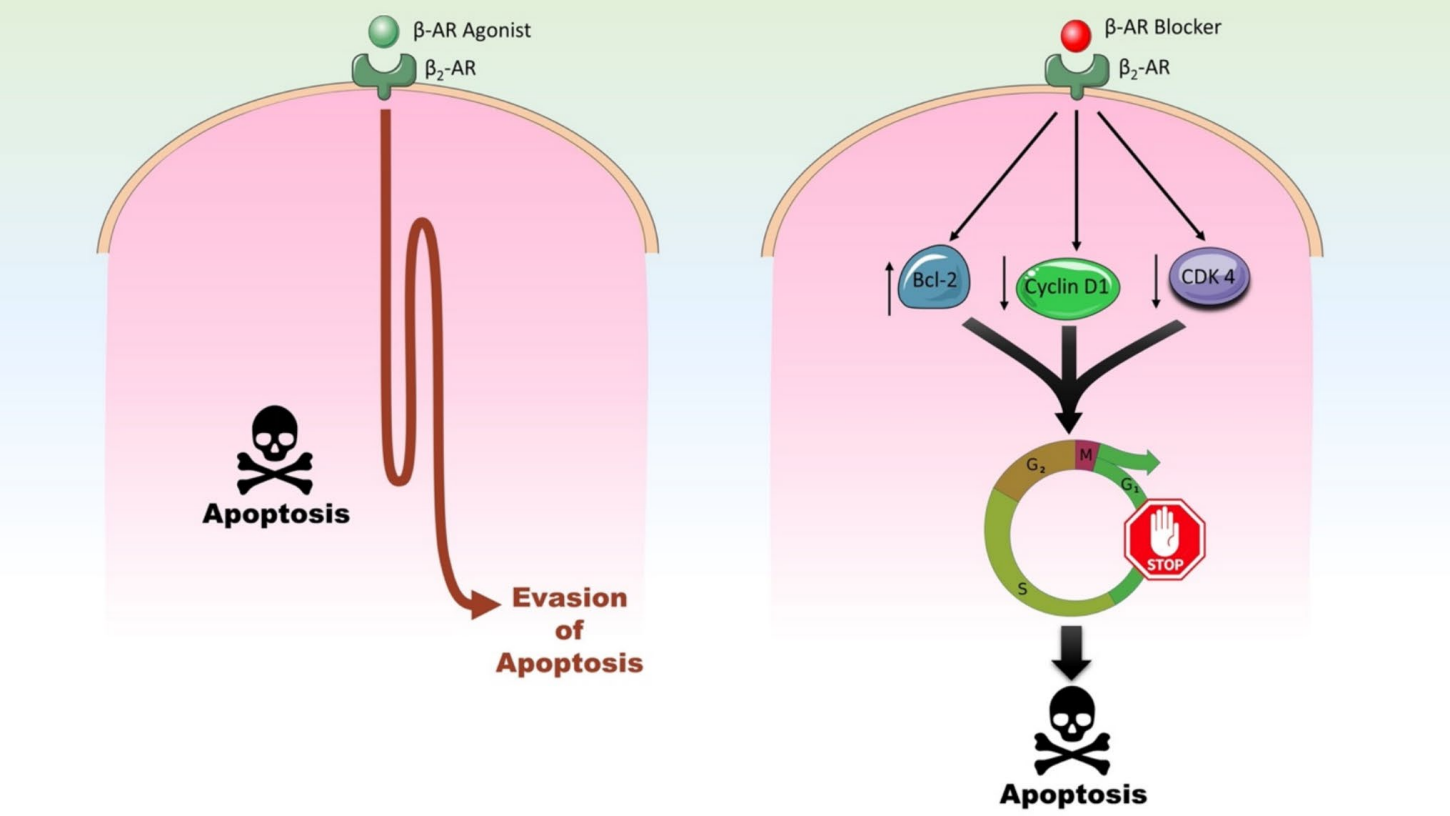
β_2_-ARs implication in the cellular evasion of apoptosis. β_2_-AR blockers may disrupt anti-apoptotic signaling pathways by upregulating pro-apoptotic Bcl-2 molecules while simultaneously downregulating cyclin D1 and CDK4, key regulators of G1 phase progression. This dual mechanisms leads to G1 cell cycle arrest and promotes the intrinsic apoptotic pathway, ultimately resulting in programmed cell death of malignant cells and tumor suppression

β-ARs have been implicated in the cellular evasion of apoptosis, a hallmark of cancer progression, including CRC, pancreatic cancer, breast cancer, prostate cancer, and melanoma (Partecke et al. 2016; Switzer et al. 2024; Kurozumi et al. 2019; Braadland et al. 2015). Epinephrine, by activating β-AR, has been shown to promote resistance to cell cycle arrest and apoptosis (Chin et al. 2016). Mechanistic studies reveal that β_2_-AR activation plays a particularly prominent role in this process. In both in vitro CRC cell models and in vivo xenografts, selective blockade of β_2_-ARs—but not β_1_-ARs—has been demonstrated to suppress cell viability, induce G1-phase cell cycle arrest, trigger apoptosis, and inhibit tumor growth. The molecular underpinnings of these effects involve the inhibition of the EGFR-Akt/ERK1/2 signaling pathway, which is critical for cell survival and proliferation ^34^. β_2_-AR antagonists have been shown to upregulate pro-apoptotic Bcl-2 family proteins, such as Bax and Bak, while simultaneously downregulating cyclin D1 and CDK4 complexes (Chin et al. 2016). This dual effect disrupts the progression of the G1 phase of the cell cycle and promotes apoptotic cell death in malignant cells (Fig. 4). These findings underscore the unique role of β_2_-ARs in apoptosis resistance compared to β_1_-ARs, further highlighting their involvement in CRC pathogenesis.

### β-ARs and CRC tumorigenesis: not independently driven, but in a context of inflammation

The role of β-ARs in CRC tumorigenesis is not independently driven but is significantly amplified in the context of inflammation. Adrenergic signaling alone exerts only weak immunosuppressive effects; however, its impact is markedly enhanced when coupled with inflammatory mediators. For instance, studies have demonstrated that the presence of epinephrine alongside tumor necrosis factor-alpha (TNF-α) or during macrophage maturation in extended cultures leads to elevated expression of cyclooxygenase-2 (COX-2), indoleamine 2,3-dioxygenase (IDO), and interleukin-10 (IL-10). These changes strongly inhibit CD8 + T-cell proliferation and interferon-gamma (IFNγ) production, thereby impairing anti-tumor immunity.

Importantly, the immunosuppressive effects of epinephrine were reversed by celecoxib, a selective COX-2 inhibitor, in both macrophage cultures and ex vivo colon cancer explant models. This underscores the role of inflammation as a critical modulator of catecholamine-induced immune suppression. Furthermore, it provides a mechanistic explanation for conflicting outcomes in studies investigating adrenergic stimulation and cancer progression. These findings highlight the potential therapeutic value of COX-2 inhibitors in mitigating inflammation-driven adrenergic signaling and its contribution to CRC progression (Cameron and Schoenfeld 2018).

The synergistic relationship between adrenergic activation and inflammation has also been explored in clinical settings. Perioperative treatment with propranolol (a non-selective β- blocker) and etodolac (a COX-2 inhibitor) has demonstrated significant improvements in tumor molecular markers among CRC patients. This combination therapy favorably modulates transcription factors such as GATA, STAT, EGR, and CREB families—key regulators of cancer-related gene expression (Haldar et al. 2020). By targeting both adrenergic signaling and inflammation, this approach offers a promising avenue for improving CRC outcomes. Nevertheless, further research is needed to fully elucidate the interplay between β-AR activation and inflammatory pathways in CRC pathogenesis.

### Stress-induced activation of β-ARs and CRC

Stress-induced activation of β-ARs represents one of the most prevalent mechanisms contributing to CRC progression. Physical or psychological stress activates the sympathetic nervous system, leading to sustained release of catecholamines such as epinephrine and norepinephrine (Dronjak and Gavrilovic 2006; Cameron and Schoenfeld 2018). While acute stress may confer adaptive benefits by enhancing survival responses (Cameron and Schoenfeld 2018; Do-Monte et al. 2015; Ghosh and Chattarji 2015), chronic stress has been documented to suppress host immune function and accelerate tumor growth across various cancers, including CRC (Cameron and Schoenfeld 2018).

Chronic stress is characterized by persistent activation of neural pathways, including the hypothalamic–pituitary–adrenal axis, resulting in prolonged secretion of stress hormones (Liu et al. 2015; Chetty et al. 2014). These hormones directly influence CRC pathogenesis by modifying cancer cell growth and proliferation through β-AR signaling (Watson et al. 2005; Palesh et al. 2007). Notably, both β_1_-AR and β_2_-AR antagonists have been shown to attenuate the carcinogenic effects of epinephrine and norepinephrine by inhibiting ERK1/2 phosphorylation (Lin et al. 2013)—a key driver of tumorigenesis.

In addition to promoting tumor growth, chronic stress undermines the efficacy of anti-cancer therapies such as sunitinib—a multi-targeted tyrosine kinase inhibitor used to mitigate angiogenesis in CRC treatment (Liu et al. 2015; Zheng et al. 2016). Norepinephrine administration or chronic restraint stress reduces sunitinib's anti-angiogenic effects by increasing vascular endothelial growth factor (VEGF) and interleukin-8 (IL-8) expression via β-AR activation (Yang et al. 2009; Thaker et al. 2007; Liu et al. 2015). Interestingly, propranolol has been reported to reverse these stress-induced effects on angiogenesis, further supporting its therapeutic potential in CRC management (Liu et al. 2015).

### Smoking-induced activation of β-ARs and CRC

Similar to stress, smoking is associated with increased epinephrine release and subsequent β-AR activation (Schuller 2019). Smoking has long been recognized as a major risk factor for various cancers, including colorectal adenocarcinoma (Jacob et al. 2018). Its pro-carcinogenic effects are mediated through complex pathways involving both direct chemical exposure and systemic adrenergic signaling.

**Fig. 5:**
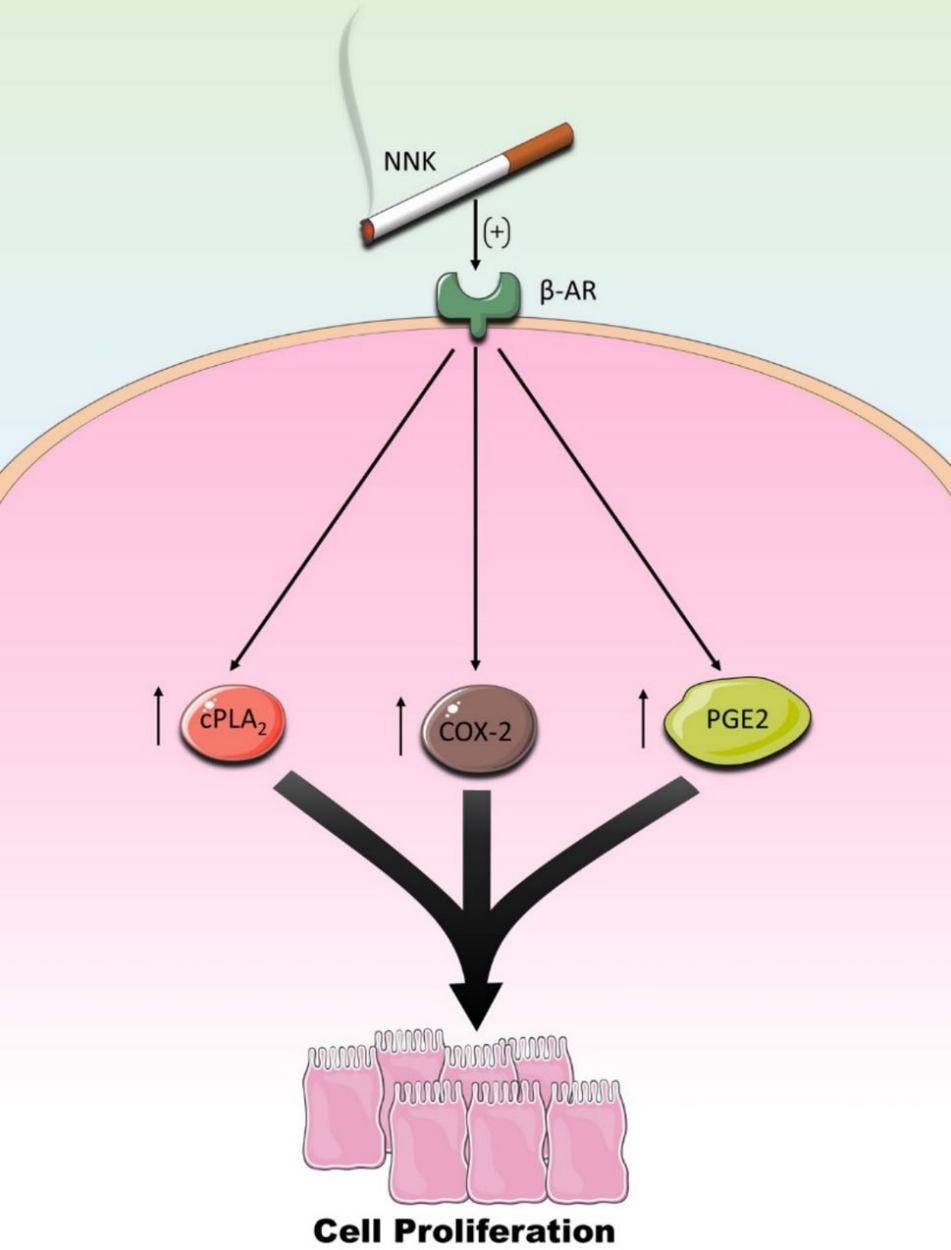
Smoking-induced activation of β-ARs and carcinogenesis. 4-(methylnitrosamino)-1-(3-pyridyl)-1-butanone (NNK), a potent tobacco-specific nitrosamine present in cigarette, smoke can activate β-ARs on cell membrane. This activation triggers a cascade of pro-carcinogenic events including increased cell proliferation through upregulating cytosolic phospholipase A_2_ (cPLA_2_) and cyclooxygenase-2 (COX-2) protein expression, which subsequently stimulates the release of prostaglandin E_2_ (PGE_2_). This inflammatory mediator promotes tumor growth by enhancing cell survival, angiogenesis and immune evasion, thereby establishing a direct mechanistic link between tobacco exposure and CRC through β-AR-mediated signaling pathways

Recent studies have linked nicotine exposure to autocrine epinephrine secretion by CRC cells, further amplifying β-AR activation (Oliveira et al. 2013). Additionally, cigarette smoke contains nitrosamine compounds such as 4-(methylnitrosamino)-1-(3-pyridyl)-1-butanone (NNK), which mimic classical β-AR agonists (Wu et al. 2005). NNK administration has been shown to increase cell proliferation via upregulation of cytosolic phospholipase A_2_ (cPLA_2_) and COX-2 protein expression while stimulating prostaglandin E_2_ (PGE_2_) release through cAMP-dependent mechanisms (Fig. 5). Importantly, these carcinogenic effects are reversed by non-selective β-blockers or selective β_2_-AR antagonists, further highlighting the contribution of smoking-induced β-AR activation to CRC progression (Wu et al. 2005).

### Prognostic role of β-ARs in CRC

**Fig. 6:**
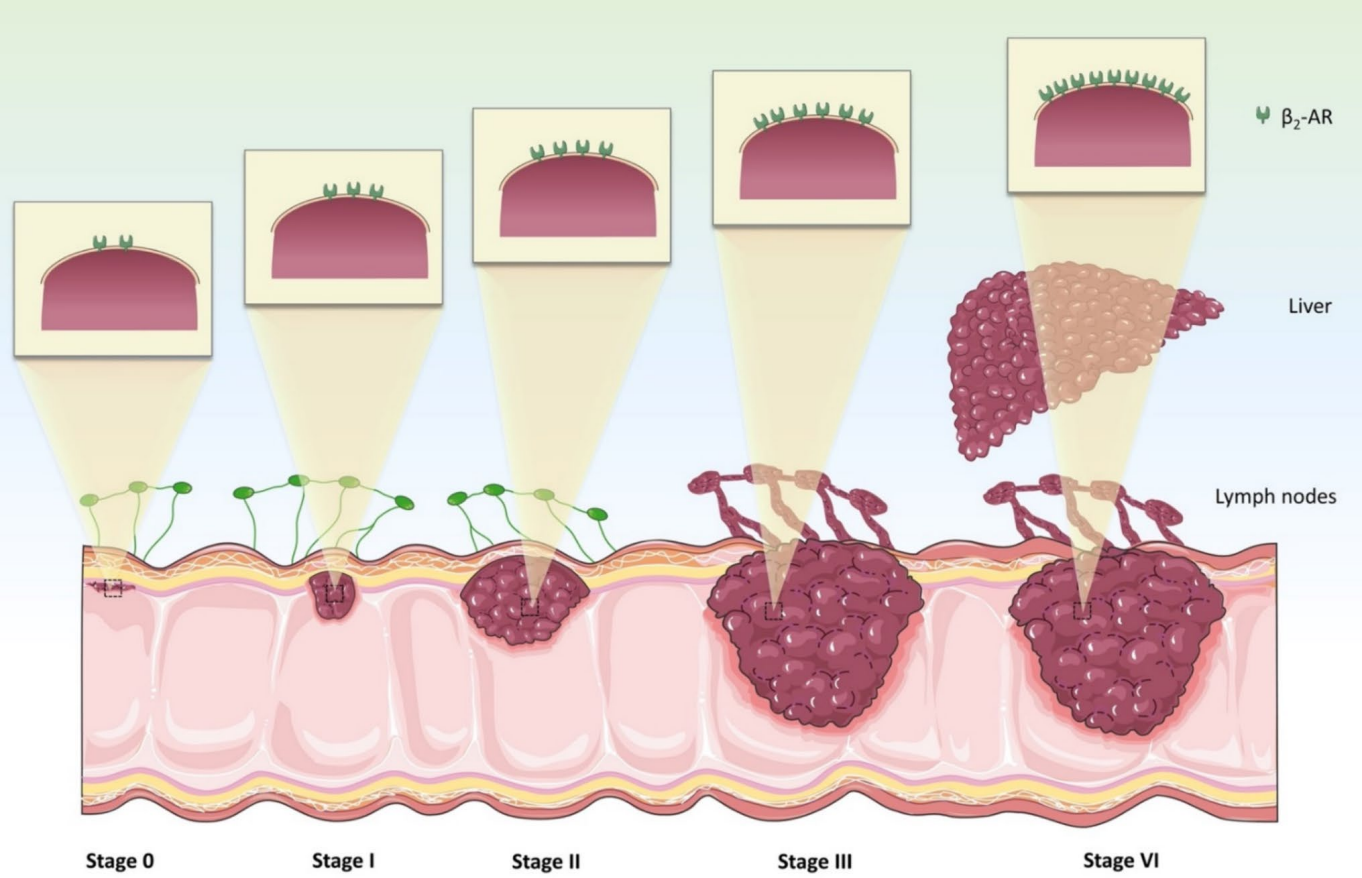
Direct relationship between the number of β_2_-AR present and CRC staging and prognosis. Immunochemical analysis of β_2_-AR expression levels in patients with stage I, II, III and IV CRC revealed a direct correlation between receptor abundance present and disease progression, with higher β_2_-ARexpression being associated with more advanced tumor stages and poorer clinical outcomes. Elevated levels of β-AR expression are associated with increased tumor aggressiveness, enhanced lymph node metastasis and accelerated tumor cell proliferation, indicating that β_2_-AR density may serve as a potential prognostic biomarker and therapeutic target. This quantitative relationship suggests that β_2_-AR expression levels could be utilized for risk stratification and treatment planning in clinical practice, with patient exhibiting high receptor expression potentially benefiting from β-blocker adjuvant therapy to improve survival outcomes

The expression of β-ARs, particularly β_2_-AR, has been implicated as a significant prognostic factor in CRC (Ogawa et al. 2020). Immunohistochemical analyses of β-AR expression across CRC stages I–IV have revealed a direct correlation between receptor levels and disease prognosis (Ogawa et al. 2020). Higher expression of β_2_-AR is associated with poorer outcomes, including increased tumor aggressiveness, lymph node metastasis, and tumor proliferation (Ogawa et al. 2020). For example, labeling β_2_-ARs with monoclonal antibodies demonstrated that their expression correlates with key tumor markers such as T (tumor size), N (lymph node involvement), and M (metastasis) factors, as well as the Ki-67 labeling index, a marker of cellular proliferation. Notably, adenoma and non-invasive adenocarcinoma patients exhibited no detectable β_2_-AR expression. However, positive expression was observed in 37%, 53%, 54%, and 75% of patients with CRC stages I, II, III, and IV, respectively (Ogawa et al. 2020). This progressive increase in β_2_-AR expression highlights its association with advanced disease stages and poor prognostic indicators (Ogawa et al. 2020) (Fig. 6). More interestingly, the level of β₂-AR expression in normal peritumoral tissue of patients with colorectal cancer has been also shown to correlate with clinicopathological features, with higher expression associated with advanced tumor stage and poor histological differentiation (Florescu et al. 2016). More recently, an animal study using CRC murine models demonstrated that β2-AR signaling promotes CRC liver metastasis by fostering an immunosuppressive microenvironment. Mechanistically, β2-AR activation enhanced the immunosuppressive activity of macrophages and myeloid-derived suppressor cells within the tumor microenvironment, thereby facilitating liver metastasis. Conversely, inhibition of β2-AR signaling reduced liver metastasis without affecting primary tumor growth (Zhang et al. 2024b). These findings emphasize the role of β_2_-ARs not only in CRC pathogenesis but also as potential biomarkers for assessing disease severity and progression.

The prognostic value of β_2_-AR extends beyond its association with tumor aggressiveness. Multivariate analyses have shown that β_2_-AR expression is an independent predictor of worse disease-free survival (DFS) and overall survival (OS) in CRC patients. Its strong association with lymphatic permeation, vascular invasion, and perineural invasion further underscores its clinical relevance. These findings suggest that β_2_-AR expression could serve as a valuable biomarker for stratifying CRC patients based on their risk profiles and guiding therapeutic decisions. Moreover, the identification of β_2_-AR as a prognostic marker opens avenues for exploring its role in treatment strategies. For instance, targeting β_2_-ARs with selective antagonists or β-blockers could potentially mitigate their oncogenic effects. Preclinical studies have already demonstrated that β-blockers like propranolol can inhibit tumor proliferation and metastasis in CRC models, supporting the rationale for integrating β-AR blockade into CRC management.

β2-AR expression levels could enable more individualized CRC treatment strategies. If validated through further research, this approach could enable clinicians to tailor therapies based on receptor expression levels. For example, patients with high β_2_-AR expression may benefit from combination therapies involving β- blockers alongside conventional treatments like chemotherapy or immunotherapy. Additionally, monitoring changes in receptor expression during treatment could provide insights into therapeutic efficacy and disease progression.

While the prognostic significance of β_2_-ARs is well-documented, evidence linking β_1_-AR expression to CRC staging and prognosis remains limited. This gap highlights the need for further studies to elucidate the potential role of other adrenergic receptor subtypes in CRC progression. Understanding these relationships could provide a more comprehensive framework for leveraging adrenergic signaling pathways in cancer therapy.

## Potential dominant role of β_2_-ARs in CRC tumorigenesis

Despite promising results from in vitro and in vivo studies demonstrating the mechanistic role of β-ARs in CRC progression, clinical translation has been hindered by inconsistent findings in human studies (Table 1). A recent pilot randomized controlled trial involving 34 CRC patients undergoing surgical resection provided clinical evidence for the therapeutic potential of perioperative β-blockade, investigating the effects of propranolol administration in combination with the COX-2 inhibitor etodolac. The intervention protocol, administered over a 20-day period beginning 5 days before surgery, was associated with a significant reduction in the transcriptional activity of tumor markers linked to malignant and metastatic potential (Haldar et al. 2020). Most compellingly, protocol-compliant analysis revealed a striking difference in disease recurrence, with zero treated patients experienced recurrence (0/11, 0%) compared to 8 out of 17 (47%) in the placebo group. Mortality was also lower in the treatment group (0/11, 0%) versus the placebo group (3/17, 17.6%), though this difference did not reach statistical significance (p = 0.151) (Ricon-Becker et al. 2023). While these findings provide important proof-of-concept for perioperative β-blockade in CRC, several methodological limitations preclude definitive conclusions. The small size inherently limits statistical power and generalizability, while the brief treatment duration may not capture the full spectrum of β-adrenergic signaling effects on tumor biology. Perhaps most significantly, the co-administration of etodolac introduce potential confounding, as COX-2 inhibition independently affects tumor prostaglandin synthesis and may synergize with β-blockade through overlapping pathways involving inflammation and angiogenesis. Additionally, the study’s single-center design and lack of long-term follow-up data limit the ability to assess durable clinical outcomes and potential late recurrences. These constraints highlight the critical need for larger, multicenter randomized controlled trials with extended follow-up periods to validate these preliminary findings and establish the independent contribution of β-blockade versus combined pharmacologic interventions in the perioperative management of CRC.

**Table 1:** Clinical trials evaluating the efficacy of β-blockers in colorectal cancer

First author, year (n)	Study design	Cancer stage	Duration	Beta blocker type	Outcome	HR (95% CI)	Limitation
Balkrishnan, 2021 (*n* = 8,025)	Retrospective	I-III	NA	Not specified	CRC-specific mortality	0.87 (0.84–0.91)	• The findings are restricted to patients aged 65 and older with stage I–III CRC enrolled in Medicare • The study relies on Medicare Part D prescription claims to estimate adherence
Musselman , 2018 (*n* = 23,076)	Retrospective	Not specified inclusion required surgical resection	53.4 ± 31.0 months	Any	CRC-specific mortality	1.14 (0.99–1.31)	• Cancer stage not available or matched for • Study limited to elderly patients (≥ 65 years)
					Overall mortality	1.06 (0.99–1.12)	
				Selective	CRC-specific mortality	1.11 (0.95–1.29)	
					Overall mortality	1.06 (0.99–1.13)	
				Non-selective	CRC-specific mortality	0.85 (0.63–1.16)	
					Overall mortality	0.85 (0.63–1.16)	
Holmes, 2013 (*n* = 722)	Retrospective	Any stage	NA	Not specified	Overall mortality	1.05 (0.93–1.18)	• Comorbidities were not controlled; non-users may have fewer comorbidities, leading to better survival • Total exposure, dosage, and compliance were not assessed • Only prescription dispensation within 1 year pre-diagnosis was considered
Springate, 2015 (*n* = 1326)	Retrospective	Not reported	Median follow-up in CPRD: 29 months Median follow-up in DIN: 30 months	Any (mostly atenolol: 73% CPRD, 75% DIN)	Overall mortality	0.85 (0.74–0.97)	• Cancer stage and treatment data were not available
Ahl, 2019 (*n* = 3187)	Retrospective	Any stage	30 days	Predominantly selective beta-1 antagonists Metoprolol (57.5%), Bisoprolol (20%), Atenolol (11%), others (11.5%)	30-day all-cause mortality	0.31 (0.20–0.47)	• Assumption made that outpatient beta-blockers were continued perioperatively • No stratification by type/class/dose of beta-blocker
Shah, 2011 (*n* = 619)	Retrospective	Not specified	Median 30 months	Predominantly selective beta-1 antagonists (atenolol 75%)	Overall mortality	1 (0.77–1.3)	• Lack of cancer stage data • Potential residual confounding
Ahl, 2020 (*n* = 22,337)	Retrospective	Any stage	90 days for short-term all-cause mortality 1 year for all-cause mortality 5 years for cancer-specific mortality	Predominantly beta-1 selective (85%)	90-day all-cause mortality	0.29 (0.24–0.35)	• The national drugs registry does not record indications for beta-blocker prescriptions, which may introduce unadjusted confounding factors
					1-year all-cause mortality	0.57 (0.52 to 0.63)	
					5-year colon cancer-specific mortality	0.8 (0.73–0.88)	
Jansen et al. 2012(*n* = 3470)	Case–control	I–IV	NA	Any	CRC risk	1.05 (0.86–1.29)	• Beta-blocker use and confounders were based on self-reports
				Selective		1.04 (0.84–1.29)	
				Non-selective		1.14 (0.75–1.75)	
Jansen et al. 2014(*n* = 1975)	Retrospective	Stages I–IV	Median 5 years	Any	Overall mortality	0.99 (0.79–1.22)	• Beta-blocker use was based on self-reports • The study did not assess beta-blocker use after diagnosis
					CRC-specific mortality	0.93 (0.71–1.21)	
					Recurrence free survival	1.04 (0.79–1.38)	
				Selective	Overall mortality	0.88 (0.7–1.10)	
					CRC-specific mortality	0.85 (0.68–1.24)	
					Progression free survival	0.92 (0.72–1.17)	
				Non-selective	Overall mortality	1.42 (0.98–2.05)	
					CRC-specific mortality	1.31 (0.82–2.08)	
					Progression-free survival	1.67 (0.99–2.79)	
Ricon-Becker et al. 2023 (*n* = 34)	Randomized controlled trial	Stage 0–III	5 years	Non-selective (propranol)	Disease-free survival	*0% (0/11) recurrence in treatment group vs 47% (8/17) in placebo (p = 0.007)	• Small sample size and single-center design limit generalizability and statistical power • Combined treatment regimen with propranolol and etodolac may not isolate the effect of beta-blockade alone
					Overall mortality	*0% (0/11) deaths in treatment vs 17.6% (3/17) in placebo (p = 0.151)	
Fiala, 2019 (*n* = 514)	Retrospective	mCRC	Median 519 days	Not specified	Progression-free survival	0.763 (0.606–0.960)	• No data on beta-blocker type, dosage, or cumulative exposure • No adjustment for changes in beta-blocker use during therapy
					Overall mortality	0.730 (0.560–0.951)	
Giampieri, 2015 (*n* = 235)	Retrospective	mCRC	NA	Not specified	Progression-free survival	1.19 (0.81–1.72)	• Lack of beta-blocker subclassification • Small sample size
					Overall mortality	1.51 (0.88–2.31)	
Kocak, 2023 (*n* = 181)	Retrospective	mCRC	Median 24.5 months (14.9–62.13)	Selective/mixed	Progression-free survival	0.66 (0.46–0.93)	• Small sample size
					Overall mortality	0.57 (0.35–0.89)	
Cui, 2019 (*n* = 890)	Retrospective	Any	Median 4 years (IQR: 1.7–6.6)	Any	Overall mortality	0.50 (0.35–0.72)	• Many patients took multiple antihypertensive drugs, limiting ability to assess monotherapy effects
					Disease-specific survival	0.50 (0.34–0.73)	
				Selective	Overall mortality	0.58 (0.41–0.83)	
					Disease-specific survival	0.63 (0.43–0.93)	
Zhang, 2022 (*n* = 5671)	Prospective	Any	28 years	Any	CRC risk	1.13 (1.01–1.27)	• Information on medication use and hypertension were self-reported • Inability to conduct dose-dependent associations
					CRC-specific mortality	0.91 (0.78–1.07)	
Weberpals, 2017 (*n* = 3572)	Retrospective	Stages I-IV	6.3 years	Any	Overall mortality	1.15 (1.05–1.26)	• Residual and unmeasured confounding • Multiple comparisons increase risk of chance findings
Ahl, 2019 (*n* = 3139)	Retrospective	Stages I-IV	1 year	Any	1-year mortality	0.66 (0.53–0.80)	• Lack of co-morbidity data • Lack of information on exact timing of BB administration, and compliance with BB therapy during hospitalization
Hicks, 2013 (*n* = 4,794)	Case–control	Stages I-IV	Median 6.3 years	Any	CRC-specific mortality	0.89 (0.76, 1.05)	• Despite adjustment for comorbidities and treatments, socioeconomic status and medication adherence could not be fully accounted for
					All-cause mortality	0.88 (0.77, 1.00)	
				Selective	CRC-specific mortality	0.90 (0.76, 1.06)	
					All-cause mortality	0.88 (0.77, 1.01)	
				Non-selective	CRC-specific mortality	0.93 (0.64, 1.34)	
					All-cause mortality	0.95 (0.72, 1.26)	
Sud, 2018 (*n* = 572)	Retrospective	mCRC	NA	Any	Progression-free survival	1.38 (0.97–1.96)	• Small sample size for BB users
Zhang, 2022 (*n* = 110,43)	Prospective	NA	Up to 28 years	NA	CRC risk	**1.13 (1.01–1.27)	• Self-reported medication use and hypertension • Data on medication dosage was not collected
					CRC-specific mortality	0.91 (0.78–1.07)	

^*^protocol-compliant analysis

^**^not significant after Bonferroni correction

In contrast, findings from observational clinical studies have been mixed, reflecting the complexity of translating preclinical mechanisms into clinical practice. A systematic review and meta-analysis of four observational studies reported a neutral association between β-blocker use and CRC risk (RR 1.00; 95% CI: 0.92–1.08). However, substantial heterogeneity was observed (p = 0.07; I2 = 58%), potentially attributable to variations in study design and geographic location (Qi et al. 2022). In contrast, another systematic review of 14 studies (*n* = 85,993) found that β-blocker use significantly improved cancer-specific mortality (*n* = 59,621; HR 0.87; 95% CI: 0.76–0.99) and 1-year overall mortality (*n* = 37,442; HR 0.54; 95% CI: 0.43–0.67), though no significant benefit was seen for overall survival (*n* = 37,975; HR 0.95; 95% CI: 0.85–1.05) (Wang et al. 2022). Similarly, a more recent meta-analysis reported that β-blocker use was associated with improved cancer-specific survival in CRC patients (*n* = 5 studies; HR 0.83; 95% CI: 0.73–0.95), but not with progression-free survival (*n* = 5; HR 0.85; 95% CI: 0.69–1.04) (Sharma et al. 2025).

One notable trial conducted in 2017 concluded that β-blockers had no effect on preventing CRC progression (Jansen et al. 2017). In this large-scale study involving 8,100 participants, both pre- and post-diagnostic use of β-blockers failed to demonstrate any significant impact on CRC outcomes, as similar mortality rates were observed between control and treatment groups (Jansen et al. 2017). A comprehensive multivariable analysis explored the effects of various β-blocker subtypes, doses, cancer sites, and stages but found no discernible benefit. However, this study had several critical limitations that may have influenced its conclusions. Notably, the majority of participants (19%) were prescribed β_1_-AR selective blockers, while only 3% received non-selective β-blockers (Jansen et al. 2017). This imbalance is particularly significant given the growing evidence implicating β_2_-ARs in CRC tumorigenesis (Ciurea et al. 2017; Ogawa et al. 2020), suggesting that the study’s reliance on predominantly β_1_-selective blockers fundamentally limits its generalizability to all β-blocker classes. Furthermore, this limitation aligns with findings from other studies that establish a stronger association between β_2_-AR expression and tumor aggressiveness, grading, and prognosis.

In addition to the overrepresentation of β_1_-AR selective blockers, other confounding factors further complicate the interpretation of these results. Participants in the trial often had comorbidities such as cardiovascular disease, which were not adequately accounted for in the analysis. The study assessed overall mortality rather than cancer-specific deaths, making it difficult to isolate the effects of β-blockers on CRC outcomes. Additionally, patients receiving β-blockers were generally older (by an average of five years), belonged to lower socioeconomic groups, and received less intensive CRC treatment compared to those in the control group (Jansen et al. 2017). These disparities likely introduced bias that could obscure any potential benefits of β-blockade in CRC management (Jansen et al. 2017).

Smaller trials have reported mixed results regarding the role of β-blockers in CRC prognosis. For instance, one study found no significant association between β-blocker use and overall CRC prognosis but identified a survival benefit for stage IV CRC patients (Jansen et al. 2014). Specifically, after adjusting for confounding factors such as age, obesity, comorbidities (e.g., hypertension or diabetes), and chemotherapy use, β-blocker treatment extended median overall survival by 18 months and CRC-specific survival by 17 months in stage IV patients (Jansen et al. 2014). Interestingly, this survival benefit was observed despite most participants using β1-AR selective blockers (24%) rather than non-selective ones (4%). In contrast, another cohort study suggested that long-term use of β-blockers (≥ 6 years) might increase progression to stage IV disease rather than prevent it (Jansen et al. 2014, 2012). This finding directly contradicts preclinical evidence showing that β-blockade inhibits tumor proliferation and metastasis. However, as with previous studies, most participants were prescribed β_1_-AR selective blockers (19%), with only a small proportion (3%) receiving non-selective agents targeting both receptor subtypes (Jansen et al. 2012).

The conflicting results from these clinical studies underscore the need to consider receptor subtype specificity when evaluating the role of adrenergic signaling in CRC pathogenesis. Preclinical research consistently demonstrates a significant role for β_2_-ARs in promoting key oncogenic processes such as proliferation, invasion, angiogenesis, and chemoresistance. For example, selective inhibition of β_2_-ARs has been shown to suppress tumor growth via downregulation of EGFR-Akt/ERK1/2 signaling pathways while inducing apoptosis through intrinsic and extrinsic mechanisms. Additionally, higher expression levels of β_2_-AR correlate with advanced tumor stages and poor prognosis in CRC patients. Given these findings, it is reasonable to speculate that the observed discrepancies in clinical studies may stem from an overreliance on β_1_-AR selective blockers rather than non-selective agents capable of targeting both receptor subtypes. This hypothesis is further supported by evidence linking elevated β_2_-AR expression to worse outcomes in CRC patients undergoing surgical resection.

To resolve these inconsistencies and better understand the role of adrenergic signaling in CRC tumorigenesis, future research should prioritize evaluating the specific contributions of β_2_-ARs. Large-scale clinical trials using non-selective or β_2_-AR specific antagonists are needed to validate their therapeutic potential. Additionally, studies should account for confounding variables such as comorbidities, socioeconomic factors, and treatment disparities to ensure more accurate assessments of drug efficacy.

## Limitations

A key limitation in interpreting the association between β-blocker use and colorectal cancer outcomes is the considerable pharmacologic heterogeneity inherent among β-blockers themselves (Ågesen et al. 2019). This indeed complicates efforts to establish definitive therapeutic relationships. Moreover, this heterogeneity manifests across multiple pharmacokinetic and pharmacodynamic dimensions that may significantly influence their biological activity within the tumor microenvironment (Cole and Sood 2012). Most notably, β-blockers exhibit marked differences in lipophilicity, a physicochemical property that fundamentally determines tissue penetration and the ability to achieve therapeutically relevant concentrations within extravascular compartments, including tumor tissues (Ripley and Saseen 2014). For example, lipophilic agents such as propranolol demonstrate superior membrane permeability and may thus achieve substantially higher extravascular concentrations compared to their hydrophilic counterparts (Kalam et al. 2020). This potentially results in more pronounced anti-tumor effects through enhanced target engagement.

The complexity of β-blocker pharmacology extends beyond simple tissue distribution to encompass critical differences in receptor affinity and selectivity profiles (Gorre and Vandekerckhove 2010). These are known to carry particular significance given the established role of β_2_-AR signaling in CRC biology (Kraboth and Kalman 2023). Many β-blockers are predominantly β_1_-AR selective; this may limit their therapeutic impact on tumor-promoting pathways that are primarily mediated through β2-ARs (Poirier and Tobe 2014). Consequently, the differential receptor targeting of various β-blockers could substantially influence their efficacy in oncologic contexts, independent of their overall β-AR blocking capacity.

Additional pharmacokinetic considerations further complicate the interpretation of existing epidemiologic data, particularly regarding the duration of action and pharmaceutical formulation characteristics of different β-blockers (Frishman and Saunders 2011). The distinction between immediate- and extended-release formulations carries important implications for achieving sustained receptor blockade, which may be necessary to effectively counter chronic stress-mediated adrenergic signaling in the tumor microenvironment (Hoffman 1998). Moreover, the temporal dynamics of β-blocker exposure introduce another layer of complexity, as chronic blockade can induce adaptive cellular responses including receptor desensitization or downregulation (Mahmood et al. 2022). This may alter therapeutic efficacy over extended treatment periods and potentially confounds long-term outcome assessments.

Beyond these well-characterized pharmacologic properties, emerging evidence suggests that β-blockers may exert off-target effects through interactions with other GPCR pathways (Yang et al. 2014) or through direct modulation of immune function (Eraky et al. 2024), mechanisms that are not routinely captured or controlled for in typical epidemiologic studies. These pleiotropic effects could either enhance or diminish the overall anti-tumor activity of β-blockers, depending on the specific agent and clinical context, yet they remain largely unexplored in current oncologic research paradigms.

The collective impact of these pharmacokinetic and pharmacodynamic differences underscores the need for more sophisticated analytical approaches in future investigations, particularly employing stratified analyses based on β-blocker class, receptor profile, and dosing regimens. Such methodologically refined studies would enable more precise characterization of the therapeutic potential of β-AR blockade in CRC and facilitate the development of evidence-based treatment strategies that account for the complex pharmacologic diversity among available β-blocker agents.

## Conclusion and perspectives

The expanding body of evidence presented in this manuscript highlights the significant therapeutic potential of β-AR modulation in CRC. Extensive preclinical studies, employing both in vitro cellular models and in vivo animal systems, have consistently demonstrated the anti-tumor effects of β-AR blockade (Lin et al. 2023; Coelho et al. 2015b; Barathova et al. 2020). These studies have further elucidated key molecular mechanisms underlying these effects, including inhibition of proliferative signaling pathways, suppression of angiogenesis and modulation of tumor cell apoptosis and metastasis. Nonetheless, it is critical to recognize the inherent limitations of such models. Indeed, they often fail to fully recapitulate the intricate complexity of the human tumor microenvironment, which encompasses not only cancer cells but also diverse non-immune stromal populations, dynamic immune cell interactions, and the heterogenous biochemical and biomechanical milieu (Arneth 2020). These limitations may, in part, account for the discrepancies between robust anti-neoplastic outcomes in preclinical settings and the more variable, sometimes inconclusive, results reported in clinical studies (Wang et al. 2022).

CRC itself represents a highly heterogenous disease characterized by diverse tumor biology, a spectrum of genetic and epigenetic alterations, and patient-specific risk factors such as environmental exposures, microbiome composition and metabolic status (Punt et al. 2017). These variables, along with differences in drug pharmacokinetics and patient adherence, adds another layer of complexity that hinders the effective translation of preclinical findings into clinical efficacy. In addition, clinical investigations evaluating β-blockers in CRC management often exhibit methodological limitations, including retrospective designs, heterogenous patient cohorts, and variable dosing regimens, all of which necessitate cautious interpretation of their conclusions (Wang et al. 2022).

An important consideration that remains insufficiently addressed is the specificity of β-AR subtypes in CRC progression and treatment response. The majority of current investigations have predominantly focused on β1-AR-selective blockers, often neglecting the significant and distinct roles of β2-AR signaling pathways in modulating tumorigenesis, immune responses, and stromal interactions within the CRC microenvironment. This selective emphasis may obscure a comprehensive understanding of β-AR blockade efficacy and mask potential therapeutic benefits achievable through dual or subtype-specific antagonism. Future studies should therefore aim to prioritize integrative approaches that carefully delineate the contributions of individual β-AR subtypes alongside the use of more physiological relevant model systems and stratified clinical trial designs, to fully capture the clinical potential of β-AR-targeted therapy in CRC. Therefore, we believe that clinical development of β-AR-directed therapeutics for CRC necessitates integration within a precision oncology paradigm, founded upon comprehensive molecular profiling and patient stratification strategies (Ahluwalia et al. 2024). Prospective randomized controlled trials are imperative to elucidate the differential oncologic efficacy of β1-selective, β2-selective, and non-selective β-blockers, incorporating stratification algorithms based on receptor expression signatures, tumor genomic landscapes, and microenvironmental determinants. The establishment of predictive biomarkers for both therapeutic response and cardiotoxicity profiles will enable adaptive clinical trial designs and personalized treatment protocols that optimize oncologic outcomes while minimizing adverse cardiovascular events.

We acknowledge that current preclinical model systems, including patient-derived organoid platforms, tumor-immune co-culture methodologies, and humanized xenograft models, will facilitate mechanistic elucidation of β-AR signaling dynamics and therapeutic mechanisms in CRC. Integration of these model systems with systems biology approaches and longitudinal multi-omics profiling will enhance mechanistic resolution and accelerate identification of novel predictive biomarkers and rational combination therapy targets. Emerging pharmacologic strategies employing third-generation β-blockers, characterized by vasodilatory properties and biased agonist/antagonist signaling profiles, represent promising therapeutic avenues due to their potential to enhance anti-tumor efficacy while attenuating cardiovascular toxicity. Furthermore, rational combinatorial approaches integrating β-AR antagonists with conventional chemotherapeutics, molecularly targeted agents, and immune checkpoint inhibitors constitute a promising strategy to overcome treatment resistance and augment anti-tumor immune surveillance.

Successful clinical translation of β-AR-targeted therapeutics in CRC requires multidisciplinary integration encompassing molecular oncology, pharmacodynamic modeling, tumor immunology, and clinical trial methodology to decipher the complex systemic and tumor-intrinsic interactions mediating β-AR signaling. Comprehensive characterization of sympathetic nervous system engagement and neurobiological crosstalk with tumor biology will inform rational therapeutic design. Integration of real-world evidence and dynamic biomarker kinetics will further refine treatment algorithms and patient selection criteria.

In closing, we argue that the clinical realization of β-AR blockade as a therapeutic modality in CRC management requires methodologically rigorous, mechanistically informed translational studies addressing receptor subtype specificity, tumor heterogeneity, and safety profiles. Through such an integrated approach, β-AR-targeted interventions may evolve into precision therapeutics capable of improving both survival outcomes and quality of life in colorectal cancer patients.

## Data Availability

Not applicable.
